# Differential utilization of primary health care services among older immigrants and Norwegians: a register-based comparative study in Norway

**DOI:** 10.1186/s12913-014-0623-0

**Published:** 2014-11-26

**Authors:** Esperanza Diaz, Bernadette N Kumar

**Affiliations:** Department of Global Public Health and Primary Care, University of Bergen, Bergen, Norway; Norwegian Centre for Minority Health Research, Oslo, Norway

**Keywords:** Immigrant, Primary care, Primary health care use, Health services, Norway

## Abstract

**Background:**

Aging in an unfamiliar landscape can pose health challenges for the growing numbers of immigrants and their health care providers. Therefore, better understanding of how different immigrant groups use Primary Health Care (PHC), and the underlying factors that explain utilization is needed to provide adequate and appropriate public health responses. Our aim is to describe and compare the use of PHC between elderly immigrants and Norwegians.

**Methods:**

Registry-based study using merged data from the National Population Register and the Norwegian Health Economics Administration database. All 50 year old or older Norwegians with both parents from Norway (1,516,012) and immigrants with both parents from abroad (89,861) registered in Norway in 2008 were included. Descriptive analyses were carried out. Immigrants were categorised according to country of origin, reason for migration and length of stay in Norway. Binary logistic regression analyses were conducted to study the utilization of PHC comparing Norwegians and immigrants, and to assess associations between utilization and both length of stay and reason for immigration, adjusting for other socioeconomic variables.

**Results:**

A higher proportion of Norwegians used PHC services compared to immigrants. While immigrants from high-income countries used PHC less than Norwegians disregarding age (OR from 0.65 to 0.92 depending on age group), they had similar number of diagnoses when in contact with PHC. Among immigrants from other countries, however, those 50 to 65 years old used PHC services more often (OR 1.22) than Norwegians and had higher comorbidity levels, but this pattern was reversed for older adults (OR 0.56 to 0.47 for 66-80 and 80+ years respectively). For all immigrants, utilization of PHC increased with longer stay in Norway and was higher for refugees (1.67 to 1.90) but lower for labour immigrants (0.33 to 0.45) compared to immigrants for family reunification. However, adjustment for education and income levels reduced most differences between groups.

**Conclusions:**

Immigrants’ lower utilization of PHC services might reflect better health among immigrants, but it could also be due to barriers to access that pose public health challenges. The heterogeneity of life courses and migration trajectories should be taken into account when developing public policies.

## Background

Ageing and migration are important socio-demographic phenomena in Europe today with obvious implications for public health policies. Aging is already becoming apparent in European populations [1], and older immigrants will also gradually represent a greater proportion in European countries like Norway [2]. Although data about migrant groups is scarce across countries because of differences in definitions and registry policies regarding immigrant background [3,4], the number of 60+ years old migrant elders in all 27 EU member states is estimated to double from seven million in 2010 to 15 million in 2015[5]. Nonetheless, elderly migrants are an extremely heterogeneous group socially, culturally and economically [5,6].

Migration itself is now recognized as a possible social determinant of health in addition to other socioeconomic factors [7]. Furthermore, in contrast with the argument that social conditions are more important than ethnicity in terms of health status of elderly immigrants, it has been argued that the ethnic and foreigner status is the determinant of the social conditions both during and after migration [4]. There are, however, contradictory findings regarding elderly immigrants and their health status and health care utilization.

Considering health status, older immigrants have generally worse health relative to host populations in most EU countries [8,9]. Also in Norway, immigrant elders struggle with more health problems than the general population, and self-perceived health is worse among immigrants than it is among Norwegians, with increasing differences with higher age [2]. Although immigrants in the USA [10,11] and in Denmark [12] appear to have lower mortality rates, other European studies have described higher mortality rates [13-15] for immigrants compared to the local-born populations. However, some studies point out the association between the pre-migration history, expressed as the wealth of the immigrant’s country of origin or the reason for migration, and mortality [12,13,15] while others conclude that the effects of current adult life socio-economic conditions on mortality are stronger than the effects of early life conditions [14,16].

Migration seems to be also a determinant of health care utilization, especially among the elderly. In Western countries, health and social welfare facilities are not easily accessible for elderly immigrants, and often their needs are not adequately or appropriately addressed [9]. Primary Health Care (PHC) utilization among elderly immigrants has been described both as higher and lower compared to the host populations of the same age. In a 11 country European sample, older immigrants reported up to 20% more utilization of health services than host populations despite adjusting for demographic characteristics [8]. In the Netherlands, immigrant elders also showed a higher self-reported use of general practitioner (GP) services, but lower use of physiotherapy and home care [17]. Most of these studies were, however, based on self-reported data, and the association between socioeconomic indicators and health outcomes was seldom based on individual data [18]. A recent Spanish register study using information from electronic health records reported lower use of PHC services among elderly immigrants compared to the national population regardless of the immigrants’ country of origin [19].

As the number of older people with immigrant background differing from the host population will continue to increase during the coming decades, a proactive response to differences in health (need) and health care utilization (access) of older migrants is becoming more compelling and should be based in empirical research. Such research should take into account the heterogeneity of the elderly migrants [6], with different ‘migrant life trajectories’, as well as other measures of socioeconomic status, to be able to address the specific needs of elderly migrants. Information from PHC is specially needed, since primary care is the first level of care where people present their health complains and where the majority of health needs are satisfied [20]. In Norway the Health Care System is tax based and all legal residents are automatically members of the national insurance with the same entitlements as citizens. PHC services include GP and Emergency Primary Care (EPC) services, which are the only ways of access to secondary care services and hospital admissions. Only very acute diseases, like heart attack, will be directly admitted at the hospital without a previous consultation at the EPC.

By means of a national register-study including all 50 years or older registered inhabitants in Norway, we aim to describe the utilization of PHC in Norway in terms of both number of consultations, diagnoses given and procedures undertaken, and to compare native Norwegians’ use of PHC services with that of different immigrant groups.

## Methods

This is a registry-based study using merged data from the National Population Register and the Norwegian Health Economics Administration database (HELFO). Personal identification numbers assigned to all Norwegian citizens and immigrants staying in Norway for at least six months were used to link the registries. Once an immigrant has obtained this identification number, access to PHC services is the same as for Norwegians.

All 50 years old or older Norwegians (born in Norway and both parents from Norway) and immigrants (born abroad and both parents from abroad) registered in Norway in 2008 were included in the study. We selected this age group based on the low life expectancy in some of the countries people migrate from [21], and because immigrants become diagnosed with chronic diseases as diabetes at a younger age than Norwegians [22,23]. The study population comprised 1,605,873 persons of whom 1,516,012 were Norwegians. Immigrants were divided according to World Bank income categories [24] depending on the gross national income (GNI) in their country of origin: low income countries (GNI 1035$ or less; 3,361 persons), lower-middle income countries (GNI 1036-4085 $; 16,057 persons), upper-middle income countries (GNI 4086-12615 $; 16,552 persons) and high income countries (GNI 12616 $ or more; 53,891 persons), and regrouped for analyses into those from high-income countries (53,891 persons) and immigrants from upper-middle, lower-middle and low income countries together (35,970 persons). For simplicity, these groups will be from now on named as “high income country (HIC) immigrants” and “other income country (OIC) immigrants”.

From the National Population Register, we obtained the following variables for all residents in Norway: gender, age, immigration category, country of origin, reason for migration (family reunification, labour, refugee or other reasons), length of stay in Norway (dichotomised for analyses by the 25^th^ percentile, 14 years), place of residence (urban vs. rural or semi-rural [25]), marital status, self-reported education level (recoded into no education, low, middle or high) and income. This register is continuously updated, and the data in this study are from 2008. Income earned in Norway was categorised in accordance with the World Health Organisation [26]: low income was defined as 60% below the median income and high income as 60% above the median income in the population studied.

The HELFO-database contains administrative claims for all patient contacts within the PHC services in Norway, including both consultations with GPs and EPC services. Relevant variables derived from this database included the number of consultations to a GP and to the EPC services for each person (both as dichotomous –yes/no- and as numerical variables), and the diagnoses received at GP’s and EPC’s consultations. Diagnoses were made based on the International Classification of Primary Care (ICPC-2) for the purpose of administrative claims, and as such can be used at the chapter level for investigation purposes [27,28]. Dichotomous variables were created for each of the ICPC-2 chapters. Each chapter variable was yes if the person had any diagnostic code in the relevant chapter in 2008, and no otherwise. In addition, the HELFO-database contains several fee codes for numerous procedures within a consultation. If the consultation lasts more than 20 minutes, a time fee is applicable. If an interpreter is present, a specific fee is applicable. However, the time fee and the interpreter fee are mutually exclusive, and cannot be used for the same consultation. There are also specific fees for conducting laboratory tests and electrocardiograms (ECG).

Descriptive analyses for Norwegians and immigrants were conducted, stratifying the groups by age categories for some analyses. Binary logistic regression analyses were conducted for the dependent dichotomous variable utilization of PHC in 2008 comparing HIC and OIC immigrants to Norwegians (reference), adjusting for different variables. Similar analyses including only immigrants were conducted to assess the association between utilization of services and both length of stay in Norway and reason for immigration. The SPSS 20.0 Software package was used for statistical analyses.

This study is part of the project “Immigrants’ health in Norway”, which was approved by the Regional Committee for Medical and Health Research Ethics and the Norwegian Data Inspectorate.

## Results

Demographic characteristics for the study population are presented in Table 1. In terms of age, percentage of women and civil status, HIC immigrants were more similar to Norwegians than OIC immigrants. OIC immigrants were younger, a higher proportion of them were men, married, living in urban areas, and had no reported formal education, although also a higher percentage among them had higher education levels compared to Norwegians. HIC immigrants had generally higher, and OIC immigrants lower income levels compared to Norwegians. Most immigrants were older than 30 years when they moved to Norway and had lived in the country for more than two decades. The reasons for immigration were first registered in Norway in 1990. However this variable is not available for Scandinavian immigrants, who comprised 32.6 percentage of HIC immigrants. Among those with registered reasons for migration at arrival, most HIC immigrants moved in order to work, while OIC immigrants were refugees or moved to Norway for family reunification.

**Table 1:** **Socio-demographic characteristics for the study population**

	**Norwegians**	**High-income country immigrants**	**Other-income country immigrants**
Numbers	1,516,012	53,891	35,970
Age, mean (SD)	65.9 (11.4)	62.8 (10.5)	59.3 (8.6)
Women, %	52.9	51.5	47.9
Urban settlement, %	62.0	76.2	86.9
Civil status, %			
Married	59.2	59.8	69.7
Widow	16.6	12.0	10.4
Single	9.1	8.2	3.9
Others	15.1	20.0	16.0
Education level, %			
No formal education	0.3	0.7	7.5
Low education	30.8	22.5	36.2
Middle education	48.2	36.5	26.6
High education	20.7	40.2	29.7
Income level, %			
No income	46.1	39.4	54.0
Low income	12.3	12.6	11.3
Middle income	32.4	37.2	29.9
High income	9.2	10.9	4.8
Age at migration, mean (SD)	n.a.	34.6 (13.1)	38.0 (12.9)
Years in Norway, mean (SD)	n.a.	29.3 (17.0)	21.1 (11.7)
Reason for immigration^1^, %			
Family reunification	n.a.	7.0	14.3
Labour immigrants	n.a.	10.0	1.9
Refugees	n.a.	1.2	26.2
Education/other	n.a.	8.9	1.1

^1^Registered from 1990, and not available for immigrants from Scandinavia. For this reason, the proportions do not add up to 100%.

Registered inhabitants 50 years old or older in Norway in 2008.

**Figure 1:**
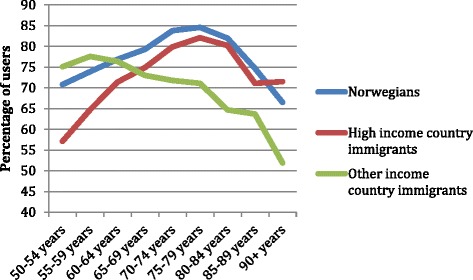
**Proportion of Primary Health Care users in 2008 by age group.** Norwegians and immigrants from high-income and other-income countries.

Figure 1 shows the proportion of Norwegians and immigrants who used PHC services (GPs and EPC together) in 2008 by age. HIC immigrants had a lower utilization of PHC compared to Norwegians, but utilization was increasingly similar in older age groups. OIC immigrants were observed to have a different pattern of utilization, with proportionally higher rates among 50 to 60 years old adults, but approximately ten per cent lower among those older than 60 years.

**Figure 2:**
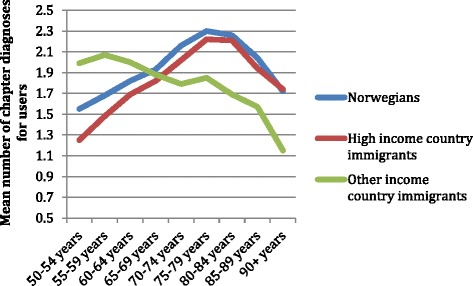
**Mean number of different diagnoses for Primary Health Care users.** Both diagnoses at the General Practitioner and at the Emergency Primary Care services included for Norwegians and immigrants by age group.

Figure 2 shows the mean number of diagnoses from different ICPC-2 chapters given to patients in 2008, according to age. Only PHC users are represented, this is to say, persons who had consulted either their GP or the EPC at least once in 2008. The number of diagnoses per user for Norwegians and HIC immigrants was similar, especially among users older than 70 years. However, respectively higher and lower comorbidity levels among the youngest and the oldest OIC immigrants compared to Norwegians were shown. To find out if these results were due to aggregated data on country of origin, we conducted separated analyses by country of origin (not shown), but this did not provide any further explanation.

Fewer immigrants compared to Norwegians used PHC services in 2008, especially among HIC immigrants (Table 2). The mean number of consultations to both the GP and the EPC, and the mean number of different diagnoses (chapters at ICPC-2) for PHC users, were lower for HIC immigrants, but higher for OIC immigrants compared to Norwegians. Regarding the characteristics of the consultations, as expected, interpreters were more often used for OIC immigrants. Long consultations at the GP took more often place among Norwegians, but fees for interpreter and long consultations are mutually exclusive. At the EPC, where interpreters are not easily available, both long consultations and interpreters were more often reported for OIC immigrants. Fewer blood tests and ECGs were ordered for immigrants compared to Norwegians at the GPs’, but more glucose related and C-reactive protein (CRP) tests were described for OIC immigrants, for whom most test were also required at the EPC. Home consultations were more frequent for Norwegians and more seldom for OIC immigrants.

**Table 2:** **Consultations to Primary Health Care services for Norwegians and immigrants in Norway in 2008**

	**Native Norwegians**	**High income country immigrants**	**Other income country immigrants**
At least one consultation to either GP or ER in 2008, %	76.6	68.3	74.9
Number of diagnoses for users of either GP or ER, mean (SD)	2.4 (1.4)	2.4 (1.4)	2.6 (1.5)
At The General Practitioner			
Number of consultations to the GP, mean (SD)	3.4 (4.2)	3.0 (4.0)	3.8 (4.4)
Long consultations at the GP, %	47.9	42.6	41.5
Use of interpreter at the GP, %	0.3	2.1	19.9
Blood tests at the GP, %	59.4	50.3	57.6
Oral glucose overload at the GP, %	0.3	0.3	0.6
Glucose test at the GP, %	21.5	17.2	25.4
CRP at the GP, %	23.7	20.3	23.9
ECG at the GP, %	8.7	7.3	8.0
At The Emergency Primary Care			
Number of consultations to the Emergency Room, mean (SD)	0.15 (0.5)	0.12 (0.5)	0.18 (0.6)
Long consultations at the EPC, %	4.9	4.1	6.0
Use of interpreter at the EPC, %	0.0	0.2	2.5
Blood test at the EPC, %	4.4	3.6	6.6
Glucose test at the EPC, %	0.5	0.5	1.8
CRP at the EPC, %	3.8	3.1	5.8
ECG at the ER, %	1.4	1.2	2.3
Home Consultations			
Home consultation at daytime, %	1.6	1.1	0.7
Home consultation out of hours, %	2.7	1.8	1.2

**Figure 3:**
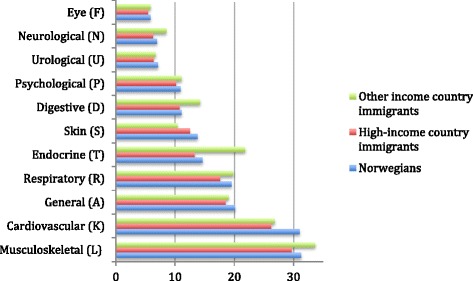
**Age and gender-adjusted proportion of the population with at least one diagnose.** Diagnoses (ICPC-2 chapters) given by either the General Practitioner or at Emergency Primary Care services.

Figure 3 represents the age and gender-adjusted percentages of Norwegians and immigrants with at least one diagnose in each of the most common ICPC-2 chapters. For most chapters, there were small differences between Norwegians and immigrants, but HIC immigrants were more seldom diagnosed. OIC immigrants more often than Norwegians had diagnoses related to endocrine (21.8 vs. 14.6% respectively) musculoskeletal (33.6 vs. 31.2%), and digestive (14.2 vs. 11.1%) problems. Norwegians had more often cardiovascular (26.7 and 30.9% for OIC immigrants and Norwegians respectively) and dermatological diagnoses (10.4 vs. 13.8%).

The utilization of PHC for immigrants compared to Norwegians is presented in Table 3 for three age groups, adjusting for age and gender (model 1), and education and income levels as socioeconomic variables in addition (model 2). A lower proportion of HIC immigrants used PCH compared to Norwegians. The same was true for OIC immigrants, with the exception of those 50 to 65 years old. On the contrary, there was an increasing gradient in utilization with age for HIC immigrants. Adjustment for education and income levels generally diminished the differences between immigrants and Norwegians.

**Table 3:** **Utilization of Primary Health Care services by age group for immigrants compared to Norwegians**

	**Age**					
	**50-65 years**		**66-80 years**		**>80 years**	
	**Model 1 OR (95% CI)**	**Model 2 OR (95% CI)**	**Model 1 OR (95% CI)**	**Model 2 OR (95% CI)**	**Model 1 OR (95% CI)**	**Model 2 OR (95% CI)**
Norwegians (reference)	1	1	1	1	1	1
Immigrants high- income countries	0.64 (0.63-0.65)	0.90 (0.87-0.92)	0.79 (0.76-0.82)	0.88 (0.84-0.92)	0.90 (0.84-0.97)	0.98 (0.91-1.05)
Immigrants other- income countries	1.20 (1.16-1.23)	1.23 (1.20-1.27)	0.55 (0.52-0.58)	0.68 (0.63-0.72)	0.46 (0.40-0.53)	0.68 (0.55-0.83)

Model 1: Adjusted for age and gender.

Model 2: Adjusted for age, gender, education and income levels.

Logistic regression analyses. Odds ratios (OR) and 95% confidence intervals (CI).

The associations between utilization of PHC and length of stay and reasons for migration are shown in Table 4, including only individuals from the two immigrant groups with information on length and reason for stay. Binary logistic regression analyses were otherwise similar to the analysis described for Table 3. Long stay in Norway (longer than 14 years) was an important predictor factor for utilization of services, but its relevance diminished, and disappeared for OIC immigrants, after adjustment for socioeconomic factors. With regards to reason for migration, labour immigrants had significant lower, and refugees higher utilization of PHC compared to immigrants moving for family reunification. These results were slightly moderated when adjusting for socioeconomic factors.

**Table 4:** **Association between length of stay in Norway and reason for migration and use of Primary Health care Services**

	**High income country immigrants**		**Other income country immigrants**	
	**Model 1 OR (95% CI)**	**Model 2 OR (95% CI)**	**Model 1 OR (95% CI)**	**Model 2 OR (95% CI)**
Individuals in the model	*53891*	*42969*	*35970*	*28936*
Short length of stay (under 14 years) (Reference)	1	1	1	1
Long length of stay (14 years or more)	2.45 (2.35-2.57)	1.32 (1.23-1.42)	1.29 (1.23-1.36)	0.99 (0.92-1.06)
Reason for migration				
Individuals in the model	*14588*	*5732*	*15676*	*10569*
Family reunification (Reference)	1	1	1	1
Labour immigrants	0.32 (0.29-0.35)	0.63 (0.52-0.76)	0.45 (0.38-0.53)	0.74 (0.57-0.97)
Refugees	1.93 (1.59-2.35)	1.55 (1.22-1.97)	1.73 (1.59-1.88)	1.32 (1.17-1.48)
Education/other	1.07 (0.97-1.17)	1.01 (0.87-1.16)	0.54 (0.44-0.67)	0.70 (0.50-0.96)

Model 1: Adjusted for age and gender.

Model 2: Adjusted for age, gender, education and income levels.

Logistic regression analyses for immigrants from high-income and other income countries. Odds ratios (OR) and 95% confidence intervals (CI).

## Discussion

### Main results

Among 50 years old or older persons living in Norway, our study reveals that a higher proportion of Norwegians use PHC services compared to immigrants. However, important differences in utilization between immigrants depending on their countries of origin are observed. While HIC immigrants used the PHC system less than Norwegians, they had similar number of diagnoses when in contact with primary care, especially the oldest ones. Among OIC immigrants, 50 to 65 years old used both the GP and the EPC more often and had higher comorbidity levels, but this pattern was reversed for older adults. For all immigrants, utilization of PHC services increased with longer stay in the host country and was higher for refugees but lower for labour immigrants compared to immigrants for family reunification. Adjustment for socioeconomic factors reduced, however, the differences between immigrants and Norwegians.

### Strengths and limitations

Our study has several strengths. Firstly, it covers the whole population in Norway aged 50 or more, eliminating self-selection bias. Secondly, the rich information on socioeconomic position and migration status allowed us to categorise immigrants into relevant groups both based on country of origin, reason for migration and length of stay in Norway. Thirdly, the data on utilization of PHC services is objective and complete as opposed to other studies relying on self-reported utilization of services.

However, our study has some limitations. Firstly, our information is only about PHC. Even though the main rule in Norway is that the GP is the only gatekeeper in the system, we do not have information about patients already using specialised care, including home consultations from secondary care to older patients. Nevertheless, PHC is by large the first point of contact, and a necessary step to secondary care also for emergencies and the share of patients attended by secondary care has earlier been reported to be similar for Norwegians and immigrants in Norway [29], whose comparison is the main aim of this study. Secondly, the diagnoses were based on ICPC-2 codes reported for administrative claims and not, for example, extracted from electronic records. This was the only possibility to obtain data on morbidity for the whole population in Norway, but it reduces the accuracy of the diagnoses, and therefore cannot be used to calculate real prevalences of diseases. They are, however, widely used and validated for research at the chapter level for comparison of populations [27,28]. Thirdly, the HELFO-database does not include consultations for individuals living in nursing homes. This will explain part of the reduction in utilization of PHC services for the oldest patients regardless of country of origin. The proportion of people living at home decreases from 96% among those 67-79 years old, to 82% and 56% for those 80 to 89 and older than 90 years old respectively [2]. Unfortunately, we have no data on the proportion of immigrants that live in nursing homes, but there are indications that immigrants might be reluctant compared to Norwegians to live away from their own homes, which would increase the differences we find among the oldest patients. Lastly, our study only takes into account consultations in Norway. Patients from other countries, especially those located near to Norway, may have travelled abroad to see a physician, and this might contribute to the differences we found between immigrants and Norwegians. Similarly, remigration to the country of origin, if not registered, might give lower utilization rates among immigrants. Recent analyses for immigrants from Asia, Africa and Latin America in Norway, however, indicate that a low proportion among the elderly move back to their countries of origin [2].

### Interpretation of results

Because of its cross-sectional nature, our study cannot provide explanations for the different utilization of PHC services among immigrants. Lower utilization would be appropriate if it reflects better health, but it could also be attributed to barriers for utilization of health services, posing a public health challenge. Identified potential barriers in the literature at patient, provider and system level include (i) patient characteristics represented by demographic and social variables, health beliefs and attitudes, personal and community enabling resources, perceived illness and personal health practices; (ii) provider characteristics such as skills and attitudes, and (iii) barriers related to the system characteristics, mainly due to the organisation of the health care system [30,31]. Older age has more recently been identified as an additional potential barrier [32]. In accordance to the largest study among elderly immigrants in Europe [8], socioeconomic variables did not completely explain disparities in utilization of services in our study, but there were substantial differences between immigrants depending on the wealth of their country of origin. Also, the inclusion of income and education levels in our analyses contributed to decrease the differences in utilization between immigrants and Norwegians. Thus, our results point to socioeconomic barriers at the patient level including both pre-migration aspects and factors at the host country that should be further studied. However, there are probably still other reasons explaining differences in utilization between immigrants and Norwegians and among the different immigrant groups.

As explained above, lower utilization could be appropriate if it reflects better health among those not attending to PHC. Some researchers would expect lower health care utilization among immigrant populations because of the initial selection of relatively healthy persons as immigrants, known as “healthy immigrant effect” [33]. This effect is thought to be strongest among recent immigrants and health is likely to converge toward that of the native-born population with longer time, adoption of new ethnocultural habits of the host country and increasing age [8]. Our results support both theories. Although utilization of PHC services increased with longer stay in Norway for all immigrants, refugees used the system more often and labour immigrants, to whom the “healthy immigrant effect” would apply the most, more seldom compared to immigrants for family reunification. It is noteworthy that the effect of the reason for migration was still significant for older immigrants who had stayed in Norway an average of more than 20 years.

Several studies also suggest that morbidity burden is the most important variable at the patient level to predict utilization of services [8,34], though it has not been studied if this affects all age groups in the same way. We cannot determine if non-users were healthy or not, but our study can give some indications regarding the morbidity of PHC users across groups. Overall, HIC immigrants had lower and OIC immigrants higher number of diagnoses (ICPC-2 chapters) compared to Norwegians. An earlier health survey among selected groups of immigrants in Norway, most of whom migrated from other than HIC, disclosed lower self rated health and higher disruption of daily live because of illness among immigrants 55 to 70 years old compared to Norwegians [29]. However, in the same study the number of self-reported consultations to the GP during the last 12 months was 7.2 for immigrants and 2.6 for Norwegians, much higher, especially for immigrants, than those reflected by objective measures in this study. This gap could be due to selection bias or recall bias in the health survey, and should be further investigated.

Similarly to a previous study among elderly patients in PHC [35], the two most common diagnoses reported in our study were cardiovascular and musculoskeletal ones. Compared to Norwegians, OIC immigrants more often had diagnoses related to endocrine, digestive and musculoskeletal problems, but less often had cardiovascular diagnoses. These results concur with a recent study of use of cardiovascular and antidiabetic drugs among immigrants in Norway (Diaz, personal communication), and with two other studies describing high prevalence of diabetes among immigrants, but underdiagnose of cardiovascular disease in this group [36,37]. However, the higher comorbidity levels for younger OIC immigrants and the lower comorbidity levels among the oldest ones in our study are hard to explain. As all the subjects in Figure 2 are PHC users, it only includes persons that have surely been in Norway at least at one point in 2008, so that the “salmon bias” effect reflecting back migration is truly not the main explanation. The concept of “health survivor” has been mainly used for workers [38], but it can also apply to the elderly among immigrants, who are well above the mean life expectancy age for some of the countries they migrate from [21], especially taking into account the tendency of immigrants to become diagnosed at a younger age than Norwegians [22,23]. Further research on health need among non-PHC users is thus necessary to determine the appropriateness of PHC utilization among immigrants, especially the eldest OIC immigrants.

Potential barriers at the professional and system levels can be partially explored through characteristics of PHC consultations. As explained in the methodological section, fees regarding consultations lasting more than 20 minutes (long consultations) and interpreter are mutually exclusive, but the GP gets a higher economical incentive when using an interpreter compared to using long time. Having this in mind, our results reflect that interpreters are used in one out of five GP consultations with OIC immigrants, and more seldom in consultations with HIC immigrants, who often speak either English or an Scandinavian language, both usually understandable for the GP. The lack of available interpreters at the EPC is thus probably the reason for a higher proportion of long consultations at the EPC for OIC immigrants, and can represent a barrier for quality of care that the physician tries to compensate by taking several laboratory tests, as previously described [39]. In contrast, in consultations with immigrants, GPs tended to order similar or less ECG and blood test, except for glucose and CRP test. According to Tran et al, patients from minority groups have worse glycaemic control than Norwegians [22], which could explain the higher use of glucose related tests. However, given that ECG should be regularly taken in patients with diabetes and cardiovascular diseases and that the prevalence of these two are probably high among several immigrant groups [37], ECGs might be underused by GPs for this population. Last, and according to the previous literature [17], the main relative differences between Norwegians and immigrants were regarding the proportion of home consultations, which were more than twice as often paid to Norwegians compared to other immigrants. This might constitute a system barrier for older immigrants that should be further investigated.

## Conclusions

In our study, a higher proportion of Norwegians 50 years old or older used PHC services compared to immigrants of the same age, but we detected differences in utilization among immigrants according to place of origin, reason for immigration and length of stay in the country. Immigrants’ lower utilization could be appropriate if it reflects better health among immigrants, but it could also be due to barriers for utilization of services, posing a public health challenge. Elderly immigrants should be actively involved in the further study of these potential barriers and in the development of appropriate services tailor made to the needs. The heterogeneity of life courses and migration trajectories should be taken into account when developing public policies.

## Abbreviations

ECG: Electrocardiogram
EPC: Emergency primary care
ICPC: International classification of primary care
GNI: Gross national income
GP: General practitioner
HELFO: Health economics administration database
HIC: High-income country
OIC: Other-income country
PHC: Primary health care
